# Sexual segregation occurs in bats within fragmented remnant woodlands in an agricultural landscape

**DOI:** 10.1002/ece3.9350

**Published:** 2022-10-01

**Authors:** Penelope C. Fialas, Lia R. V. Gilmour, Sophie Vickress, Emma Underwood, Carol A. Williams, Helen Miller, Paul R. Lintott

**Affiliations:** ^1^ School of Applied Sciences University of the West of England Bristol UK; ^2^ College of Life and Environmental Sciences University of Exeter Exeter UK; ^3^ Bat Conservation Trust London UK; ^4^ Kingston University London Kingston upon Thames UK; ^5^ Botanic Gardens Conservation International Richmond UK

**Keywords:** chiroptera, citizen science, landscape connectivity, sexual segregation

## Abstract

Species‐specific responses to landscape configuration and landscape composition have been studied extensively. However, little work has been done to compare intraspecific differences in habitat preferences. Bats have potential as good bioindicator taxa in woodland habitats. Therefore, studying sex differences in responses to woodland and the wider landscape can allow us to gain insight into the relative importance of these habitats for both bats and other taxa. In this study, we aimed to test the predictions that (i) habitat type and connectivity will influence the probability of recording female bats in woodlands and (ii) sex differences in response to habitat type and connectivity will be species‐specific. Bat capture data was collected in 206 woodlands over 3 years in England. The probability of detecting females relative to males was modeled in response to a range of woodland characteristics and landscape metrics for six bat species. We recorded sex differences in responses to landscape features in three species. We found a higher probability of capturing female *Myotis nattereri* in woodlands that were surrounded by a higher proportion of improved grasslands, whereas female *Myotis mystacinus* were less likely to be recorded in woodlands surrounded by semi‐natural vegetation. Female *Plecotus auritus* were more likely to be recorded in isolated woodlands with less connectivity to other woodlands and where agriculture dominated the surrounding landscape. Our findings indicate that sexual segregation occurs across several UK bat species in response to landscape connectivity and composition. Sexual segregation in response to landscape characteristics in bats should therefore be an important consideration in the management of fragmented agricultural landscapes.

## Graphical abstract

The probability of detecting females relative to males was modeled in response to a range of woodland characteristics and landscape metrics for six bat species. We recorded sex differences in responses to landscape features in three species. Our findings indicate that sexual segregation occurs across several UK bat species in response to landscape connectivity and composition.

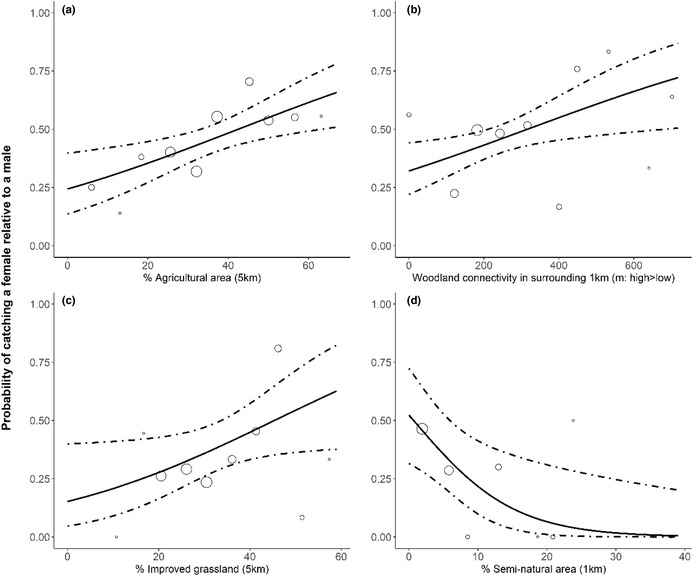

## 1 INTRODUCTION

Agricultural expansion is considered one of the main drivers of habitat loss and fragmentation and is a global threat to biodiversity (Ciccarese et al., 2012; Haddad et al., 2015). Forest systems and woodland environments support a wealth of biodiversity worldwide yet are severely affected by human encroachment and depletion of resources (Haddad et al., 2015). Global forest cover has been reduced by ca. 50% in the last three centuries (Ramankutty & Foley, 1999) and much of the remnant woodland that survives is highly fragmented and reduced in size, which has major implications on species patterns of habitat use. Species‐level responses to woodland fragmentation have been studied on a broad scale, in a wide range of taxa (Fuentes‐Montemayor et al., 2019). However, there has been relatively little consideration of the importance of intraspecific responses to landscape structure and connectivity, despite its importance for the dynamics and long‐term persistence of ecological communities (e.g. Lintott, Bunnefeld, et al., 2014).

Habitat segregation between sexes is taxonomically widespread and occurs because of differences in parental care (Lucass et al., 2016), antipredator behavior (Curlis et al., 2016), and responses to stress (Small & Schoech, 2015). Sexual differences in animal behavior can result in habitat segregation and have potential to adversely impact the sustainability of fragmented populations (e.g. Angell et al., 2013; Nardone et al., 2015; Senior et al., 2005). Understanding sex differences in responses to local and landscape factors is therefore important in determining the long‐term health of fragmented populations and to ensure that conservation actions can be targeted efficiently and effectively.

Sexual segregation in bats can occur in roost selection and within the roost (Senior et al., 2005), whilst foraging (Lintott, Bunnefeld, et al., 2014), and during migration (Fleming & Eby, 2003). The high energetic demands of pregnancy and lactation can restrict females to preferentially foraging within high quality habitats (e.g. woodland), thereby limiting their use of marginal upland habitat (Senior et al., 2005), arable land (Mackie & Racey, 2007) and woodlands within an urban landscape (Lintott, Bunnefeld, et al., 2014). Therefore, sex differences in response to habitat type and configuration can help infer where targeted habitat management to enhance existing habitat (e.g. retention of standing trees) and habitat creation should be focused to best conserve fragmented landscapes.

Most bat species have some reliance on woodland for at least part of their life‐history and others are completely reliant on these habitats for roosting, foraging, commuting and reproduction (Boughey et al., 2011; Fuentes‐Montemayor et al., 2013; Lacki et al., 2007). In the UK, woodland is highly fragmented with patches of varying ages, sizes, and degrees of isolation scattered through an agricultural landscape (Fuentes‐Montemayor et al., 2013). Intraspecific differences in habitat requirements in bats may restrict the distribution of sexes within agricultural landscapes, however, to our knowledge, few studies to date have assessed this in multiple species (e.g. Hill & Greenaway, 2006; Miller, 2012).

In this study, we use data collected for the Bat Conservation Trust's Bechstein's Bat Survey, an ambitious citizen science project that aimed to expand the known range of a rare woodland specialist bat (*Myotis bechsteinii*) in the UK (Miller, 2012). Bat capture data for a range of bat species were collected under Natural England bat license in 206 woodlands, over three years by experienced Bat Conservation Trust volunteers. Using this capture data, we aimed to assess sex differences in the use of fragmented woodland landscapes.

Here we compare sex differences in six UK bat species in response to a range of woodland characteristics and landscape metrics. We aimed to test the predictions that the probability of finding a female compared with a male bat will be higher in woodlands that (1) are well connected in the landscape, (2) represent a higher quality habitat (e.g. higher percentage canopy cover of native broadleaf trees) and (3) are surrounded by a higher quality habitat (e.g. semi‐natural areas such as unimproved grassland). We also predicted that these sex differences will be species‐specific, with lower mobility species (e.g. *Plecotus auritus*, and *M. bechsteinii*) more likely to have reduced tolerance to habitat fragmentation and lower quality woodland and surrounding habitat than generalist species (e.g. *Pipistrellus pipistrellus*).

## 2 MATERIAL AND METHODS

### 2.1 Site selection and woodland surveys

**FIGURE 1:**
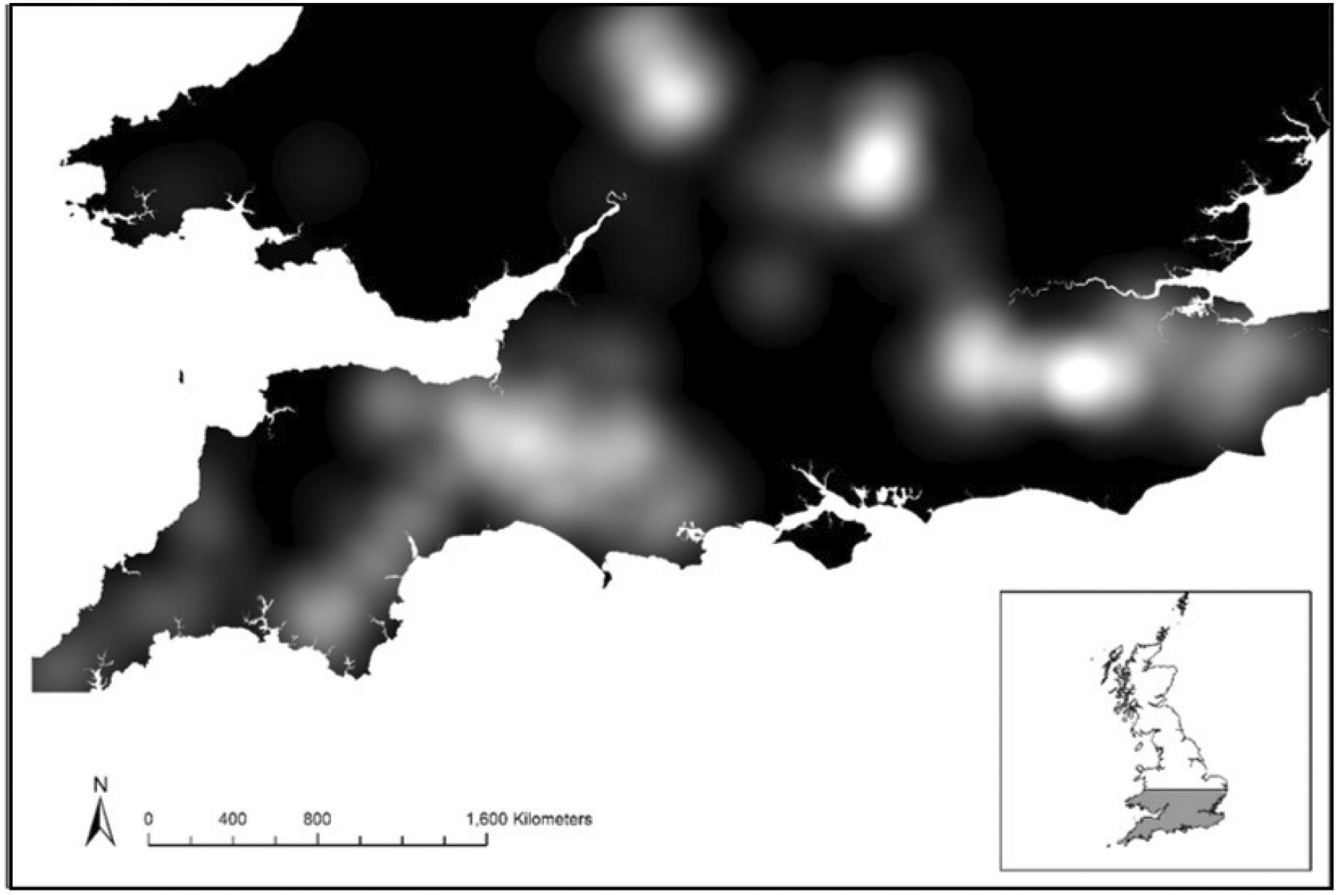
Kernel density map of woodlands meeting the selection criteria of the study. Black indicates absence of woodland sites and lighter shades indicate areas with woodland sites surveyed.

Sites were selected and surveyed once as part of the Bat Conservation Trust's Bechstein's Bat Survey (Miller, 2012). Woodlands were selected within the known UK distribution of *M. bechsteinii* which spans southern England and South Wales (Figure 1), with the following counties prioritized for surveying effort: Dorset, Gloucestershire and Somerset. Each county was divided in 10 km squares following the Ordnance Survey grid system and a single woodland‐site in each 10 km square was selected according to six key criteria used to maximize the chance of detecting *M. bechsteinii*, including a high proportion of native broadleaf trees and a well‐developed native understory layer (Appendix S1; see Miller, 2012). In squares where more than one woodland met the initial criteria, additional stratification occurred (Appendix S2). Each woodland chosen to be included in the project was visited to record the percentage of canopy cover and understory cover around each trapping location and across the woodland as a whole, the dominant canopy and understory species, and the obvious presence of woodland management in place (e.g. grazing animals, and coppicing).

### 2.2 Bat surveys

Surveys were carried out in the summer months (May–September) from 2008–2011 by 10 trained local bat groups, supervised by licensed bat workers. Two trapping locations were selected for each woodland, an average of 331.47 m apart (range 200.07–880.64 m), and an average distance of 120 m (range 20–800 m) from the woodland and 10 m from rides, glades and other open areas, in locations designed to maximize the chance of capturing *M. bechsteinii*. A harp trap was placed at each trapping location alongside an acoustic lure (Sussex AutoBat) to increase trapping success (Appendix S3; Lintott, Fuentes‐Montemayor, et al., 2014). Harp traps were used, in contrast to mist nets, to minimize the removal time and therefore stress to bats on extraction by volunteers that often had less experience of extraction of bats from netting. Previous studies (e.g. Fukui et al., 2001) have demonstrated similar levels of capture efficiency between harp traps and mist nets in broad‐leaved woodland. Male and female bats can differ in their attraction to acoustic lures, with males of some species caught more often than females (Gilmour, 2014; Lintott, Fuentes‐Montemayor, et al., 2014; Miller, 2012). However, this is unlikely to bias our results, as we specified probability of capturing females compared with males as a relative response variable in our models (see 2.4 Statistical analysis). Surveys commenced one hour after civil twilight and lasted for 1.5 hours to maximize the trapping time when *M. bechsteinii* females are most responsive to an acoustic lure (following Hill & Greenaway, 2005). All caught bats were identified to species/genera, aged, sexed, weighed, measured and assessed for breeding condition. Species identified as *Myotis mystacinus* may potentially have been one of the co‐occurring cryptic whiskered bat species (*M. brandtii* or *M. alcathoe*). Therefore, although we refer to *M. mystacinus* throughout, findings should be interpreted at the species group level rather than for solely (*M. mystacinus*). Temperature was also recorded at the start of each survey.

### 2.3 Landscape analysis

We used ArcGIS 10.6 (ESRI, Redlands, California) to determine the central point of each woodland patch (by plotting trap locations) and created buffers around each patch of 500, 1000, 2000, 3000, 4000, and 5000 m radius, to incorporate both site‐specific characteristics (500 m) and the core sustenance zones for most UK bat species. Feature classes from the Land Cover Map 2007 were reclassified into a series of discrete biotope types following Morton et al. (2011). Biotope types included (i) broadleaved/mixed woodland; (ii) coniferous woodland, (iii) agricultural areas (i.e., arable and horticulture), (iv) improved grasslands, (v) semi‐natural vegetation (e.g. unimproved grassland types), (vi) heath areas (e.g. heather grassland and scrub), (vii) mountain bog, (viii) saltwater, (ix) freshwater, (x) costal habitat and (xi) urban areas. Biotope types with sufficient extent and variation (Appendix S4) were used to calculate a selection of compositional and configurational landscape metrics for each buffer scale of each site (Table 1). The percentage of land covered by each biotope, woodland Euclidean nearest neighbor (ENN; a metric commonly used to assess woodland connectivity, e.g. Fuentes‐Montemayor et al., 2013), woodland edge density, and Shannon's diversity index (a measure of landscape heterogeneity) were calculated for each buffer scale using Fragstats v.4.2 (McGarigal et al., 2012). Additionally, we used the National Forest Inventory to measure the area and shape of each woodland. Shape was calculated using the perimeter of the woodland divided by the minimum perimeter possible for a maximally compact patch of the same area (which equals 1 when the patch is maximally compact and increases as shapes becomes irregular, McGarigal et al., 2012). The Ancient Woodland Inventory (Forestry Commission, 2011) was used to categorize the age of each woodland into either (i) old (remnant woodland existing since at least 1880), (ii) modern (post 1880), or (iii) mixed aged woodland.

**TABLE 1:** Description of landscape variables (compositional and configurational) and local variables used for statistical analysis.

Compositional landscape variables	Configurational landscape variables	Local habitat variables
% of Each Biotope	Woodland Edge Density (ED)	Canopy cover (%)
Shannon's Diversity Index (SHDI)	Mean value of Euclidean nearest neighbor distances between all woodland patches (ENN)	Understory cover (%) Distance to nearest freshwater
Mean woodland patch area	Mean value of Euclidean nearest neighbor distances between all broadleaved woodland patches (ENN)	Distance to nearest edge
	Shape woodland patch index	

### 2.4 Statistical analyses

We performed general linear mixed‐effects models (GLMMs) with binomial error distribution and a logit link to quantify the influence of woodland characteristics and landscape metrics on the probability of capturing females compared with males for a range of UK bat species. Only sites where at least one individual was captured were included as responses variables for each species. To assess the relative effects of the explanatory variables on females compared with males, the models were run with the proportion of females to males as the response variable, with site included as a random factor. The following predictor variables were included in the model: (i) local‐woodland metrics: (ii) landscape configuration metrics and (iii) landscape composition metrics. Temperature and date were included in all models as covariates. To avoid multicollinearity and overfitting the final models, we undertook a preliminary assessment of which key landscape predictors should be included in each final model. We used GLMMs for the proportion of females to males per species with single landscape parameters (at each spatial scale) to identify the landscape predictors with the highest *R* ^2^ value. If several landscape parameters were of equal importance (i.e. <5% difference between *R* ^2^ values), they were all selected, if they were not strongly correlated. We centered and standardized continuous predictor variables, following Schielzeth (2010) to enable the direct comparison of the effect size of estimated coefficients. We then calculated the variance inflation factor (VIF) for each final model and variables that showed VIF > 3 were excluded for further analysis (Appendix S5; Zuur et al., 2010).

Statistical analyses were conducted in R version 3.4.1 (R Core Team, 2016) within RStudio v0.99.484, using the lme4 (Bates et al., 2015), effects (Fox, 2003), ggplot2 packages (Wickham, 2016). All predictor variables were tested for collinearity; however, none were considered to be strongly correlated when using a Pearson correlation coefficient of greater than 0.6 and a *p*‐value of <.05.

We compared a series of candidate mixed models containing all possible predictor variable combinations using the *dredge* function from the MuMIn package (Bartoń, 2016).

We adopted an information theoretical model‐selection approach (Grueber et al., 2011) to compare a series of candidate mixed models which contained all possible predictor variable combinations. Models were ranked on Akaike's Information Criterion with corrections for small sample sizes using the MuMIN package (Bartoń, 2016). For each competing model, we used multimodel interference (averaging ΔAICc < 2) to obtain an averaged regression coefficient and to determine the relative importance of each fixed effect following Guthery et al. (2003). Models were validated by using DHARMa package (Hartig, 2022) and visual examination of residuals plots following Zuur et al. (2010). Residual spatial autocorrelation in the final GLMMs models was inspected by means of Mantel test (Appendix S6).

## 3 RESULTS

A total of 873 bats within 206 woodland sites were captured (Table 2). The dominant species that were caught were *P. auritus* (41% of all bats caught), *Myotis nattereri* (13%), *M. mystacinus* (10%), *M. bechsteinii* (7%), *Pipistrellus pipistrellus* (9%) and *P. pygmaeus* (8%). Additional species were captured (Table 2), however, these were of insufficient quantity to include within subsequent analysis. Additionally, juveniles of all species were found in an insufficient number of sites and therefore excluded from further analysis. Females represented 40% (386) of all captures, with *M. bechsteinii* being the only species where females were found more often than males (52%).

**TABLE 2:** Bat species/genera detected in Bat Conservation Trust's Bechstein's Bat Survey. The total number of Juveniles captured are noted in brackets.

Species	Number of sites detected	Adult females (juveniles)	Adult males (juveniles)	Total
*Plecotus auritus*	156	174 (14)	201 (15)	404
*Myotis nattereri*	86	51 (2)	75 (3)	131
*Myotis mystacinus*	50	34 (4)	54 (5)	97
*Pipistrellus pipistrellus*	53	37 (7)	33 (10)	87
*Pipistrellus pygmaeus*	52	18 (4)	52 (1)	75
*Myotis bechsteinii*	37	26 (3)	25 (2)	56
*Myotis daubentonii*	23–24	7 (2)	21 (0)	30
*Myotis mystacinus/brandtii*	21	14 (1)	9 (1)	25
*Myotis mystacinus/brandtii/alcathoe*	9	6 (1)	12 (2)	21
*Myotis brandtii*	14	9 (0)	7 (1)	17
*Nyctalus noctula*	14	3 (3)	6 (3)	15
*Barbastella barbastellus*	9	4 (0)	5 (0)	9
*Pipistrellus* spp.	4	0 (0)	4 (0)	4
*Eptesicus serotinus*	4	1 (1)	2 (0)	4
*Myotis alcathoe*	2	1 (0)	1 (0)	2
*Rhinolophus hipposideros*	2	1 (0)	1 (0)	2
*Nyctalus leisleri*	1	0 (0)	1 (0)	1
*Rhinolophus ferrumequinum*	1	0 (0)	1 (0)	1
Total		386 (42)	510 (43)	981

The importance of local, configurational and compositional landscape variables was species‐specific and differed between the sexes for *P. auritus, M. nattereri* and *M. mystacinus* (Table 3; see Appendices S7 and S8 for final model statistics and model averaging results). No sex difference in habitat use was found for either *P. pipistrellus*, *P. pygmaeus* or *M. bechsteinii*.

**TABLE 3:** Statistical significance of landscape variables influencing the probability of finding a female bat relative to a male. Standardized, model‐averaged parameter estimates with associated unconditional standards errors (SE), *z*‐values, *p*‐values, significance (sig) of each and marginal *R* ^2^ for each response variable of the most parsimonious GLMMs (ΔAICc < 2) are given for each model. The full description of the most parsimonious models can be found in Appendices S7 and S8. Significance is indicated using an asterisk where * represents *p* < .05 and ** represents *p* < .01.

Response variable	Independent variable	Estimate	SE	Lower 95% CI	Upper 95% CI	*z* value	*p*	Sig	*R* ^2^
*Plecotus auritus*	% Arable area (5 km)	0.43	0.14	0.15	0.71	3.00	**<.01**	**	0.14
	Connectivity to any Woodland (1 km)	0.30	0.12	0.07	0.53	2.54	**.01**	*	
*Myotis nattereri*	% Improved grassland (5 km)	0.54	0.23	0.09	1.00	2.34	**.02**	*	0.16
*Myotis mystacinus*	Semi‐natural area % (1 km)	−1.01	0.44	−1.87	−0.15	2.31	**.02**	*	*0.29*

**FIGURE 2:**
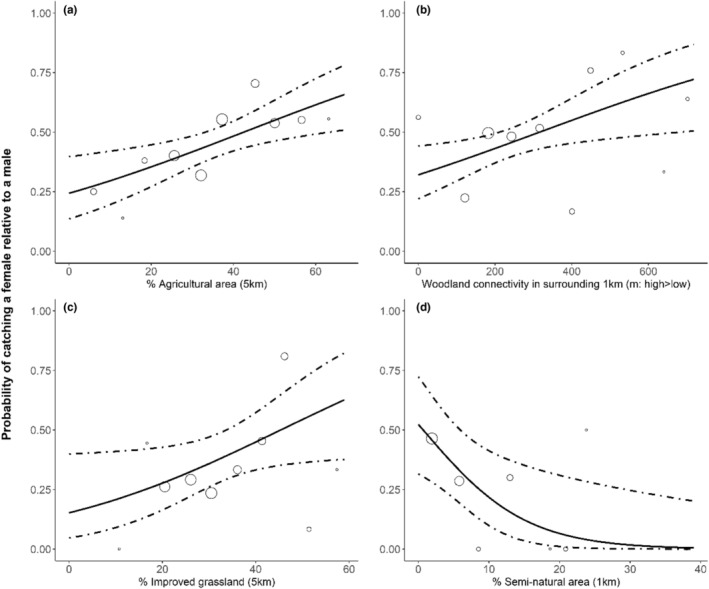
Predicted probability of finding a female relative to a male for (a, b) *Plecotus auritus*; (c) *Myotis nattereri* and (d) *M. mystacinus*, in relation to landscape variables in a fragmented woodland (agricultural area, woodland connectivity, improved grassland and semi‐natural area). Woodland connectivity (b) is calculated using Euclidean nearest neighbor distance (ENN), with a lower ENN value representing a more highly connected woodland and a higher ENN value a more isolated woodland. Model predictions from GLMMs and associated 95% confidence intervals are represented by the solid lines and dashed lines, respectively. Raw data on the proportion of females are represented with open circles with size being proportional to the total number of females.

The probability of finding female *P. auritus* was enhanced by the proportion of agricultural area in the surrounding 5 km. Based on the estimated coefficients in Table 3, the predicted probability of capturing a female compared with a male was 0.35 (0.27–0.44) in woodland surrounded by relatively little agriculture (20%), 0.48 (0.42–0.54) in woodland with moderate amount of agricultural area (40%) and 0.60 (0.48–0.71) in woodlands with high amount of agriculture area (60%; Figure 2a). Woodland isolation (ENN) in the surrounding 1 km also had a positive influence on the probability of capturing a female compared with a male, which was 0.43 (0.37–0.49) in isolated woodland, whilst there was little difference in the probability of capturing females (0.54; 0.45–0.64) compared with males (0.46; 0.40–0.52) in moderately connected woodland (Figure 2b).

Sex differences in *M. nattereri* habitat use were determined by the proportion of improved grassland in the surrounding landscape (5 km). The probability of capturing a female compared with males increased in woodlands with landscapes containing higher levels of improved grassland in the surrounding 5 km (0.62; 0.37–0.82). In woodlands with less improved grassland area, the probability to capture a female *M. nattereri* compared with males was 0.27 (0.16–0.42; Figure 2c).

Landscapes with higher percentages of semi‐natural vegetation (e.g. rough grassland) in the surrounding 1 km of a woodland negatively impacted the probability of capturing *M. mystacinus* females compared with males. Based on the estimated coefficients of Table 3, the probability of capturing females compared with males in woodlands with low levels of semi‐natural vegetation was (0.21; 0.09–0.41), whereas this reduced to 0.01 (0.003–0.22) in woodlands surrounded by higher levels of semi‐natural vegetation (Figure 2d).

## 4 DISCUSSION

This is the first study that we are aware of to present species‐specific sex differences in bat species occurring in Europe in response to habitat type and configuration in a fragmented rural woodland context. As predicted, we found that three bat species demonstrate sex differences in habitat use due to the composition and configuration of the landscape. Both woodland composition and forest fragmentation are known to influence bat abundance, with the extent of the impact varying between species (e.g. Ethier & Fahrig, 2011). However, this study highlights that male and female bats also respond differently to fragmented woodland landscapes. Our findings therefore indicate the crucial importance of conserving woodland patches within the agricultural landscape as females within several species are found more frequently within fragments surrounded by a relatively inhospitable landscape. Our results indicate that the quality of the habitat surrounding a woodland can affect how female bats of three relatively generalist species utilize woodlands and the surrounding landscape compared with males. *Plecotus auritus*, *M. nattereri* and *M. mystacinus* all use both woodland and surrounding landscape features such as hedgerows and grasslands for foraging (Arlettaz, 1996; Berge, 2007; Buckley et al., 2013; Murphy et al., 2012; Swift & Racey, 2002). However, our results indicate that if the quality of the surrounding landscape is poor, female bats are restricted to foraging in isolated woodland patches, which may represent islands of productive high‐quality habitat within relatively lower quality agricultural landscapes (e.g. Fuentes‐Montemayor et al., 2013). It may also be the case that even if an isolated patch of woodland is sub‐optimal for a population and relatively low in productivity, sexual segregation may still exist, as the woodland habitat is still preferable to females over the surrounding landscape. This preference may also be more pronounced in species that rely more heavily on woodland habitats for foraging, roosting and socializing, for example *P. auritus*.

We predicted that the probability of finding females compared with male bats will be higher in woodlands that are well connected within a landscape. Female bats have higher energy demands during pregnancy and lactation, and a shorter period to accumulate sufficient fat to survive hibernation than males, so will often forage closer to the roost and in higher quality habitats (e.g. native, well‐connected woodland with vegetation characteristics that support foraging efficiency; Lintott, Bunnefeld, et al., 2014) in order to meet their resource demands (Altringham et al., 2005; Fleming & Eby, 2003; Lintott, Bunnefeld, et al., 2014; Mackie & Racey, 2007; Senior et al., 2005). Our results indicate that female *P. auritus* bats were more likely to be recorded than males in woodlands surrounded by agricultural areas and those with a lower degree of connectivity to other woodlands. The observed sexual segregation in *P. auritus* may therefore be due to competition for insect prey, with males being forced out of woodland areas, which represent a higher quality habitat than the surrounding grassland.

We predicted that there would be an increased probability of encountering females in woodland surrounded by higher quality habitat. But contrary to our predictions, female *M. nattereri* were more likely found in woodlands where the surrounding habitat was lower quality (e.g. improved grasslands). However, when the habitat surrounding woodlands was of higher quality (e.g. unimproved grasslands), female *M. mystacinus*, were less likely to be found in woodlands compared with males, probably in favor of higher quality surrounding semi‐natural habitat (e.g. unimproved grasslands). Improved grasslands are often actively managed habitats that are either grazed or improved with fertilizer and are distinguished from unimproved grasslands by their relatively low diversity of native plant species (Fuller, 1987; Woodcock et al., 2009). Due to the dominance of grass species in these habitats, insect abundance and diversity is also likely to be low (Woodcock et al., 2009), therefore, providing less available prey for bats than semi‐natural habitats, such as unimproved grasslands (Razgour et al., 2011). The results for *M. mystacinus* support our findings for *P. auritus* and *M. nattereri*, as when the surrounding habitat represents a better foraging resource, females are less constrained to woodland patches.

We predicted that higher quality habitat (e.g. higher percentage canopy cover of native broadleaf trees) would increase the probability of finding females relative to males, however, landscape factors appear to be a much stronger driver of sexual segregation than local woodland factors. This likely reflects that the woodlands included in the study were selected to increase the probability of finding bats therefore all had a high proportion of native broadleaf trees and a well‐developed native understory layer (Miller, 2012). From a conservation perspective, it highlights the importance of a landscape approach (e.g. Sayer et al., 2013) given that interventions at a woodland scale may be hindered by the composition and connectivity of the wider matrix. It also highlights how sexual segregation in some bat species can bioindicate the relative quality of different habitats in a fragmented woodland context and highlight where habitat improvement is needed for the benefit of the local ecosystem. For example, isolated woodland patches, surrounded by poorer quality habitat may be important to conserve, but improving the landscape surrounding those patches may be equally as important.

Roost availability within woodlands may also limit bat species' utilization of the landscape (Boughey et al., 2011). Therefore, these findings may have worrying consequences for bat populations relying on isolated remnant woodland habitats. For example, the carrying capacity of maternity roosts in isolated woodland patches may be limited by the foraging resources available, potentially leading to increased female mortality rates and skewed sex ratios. In addition to this, declining populations of key insect prey, such as moths (Fox et al., 2014), could place even greater pressures on populations restricted to woodland habitats.

It is worth noting that sexual segregation was not found in several species including the woodland specialist *M. bechsteinii*. Female *M. bechsteinii* bats are thought to be more demanding in their habitat requirements than males, though this can depend on reproductive status, with non‐breeding females often found in sub‐optimal habitats (Hill & Greenaway, 2006, 2008; Miller, 2012). However, *M. bechsteinii* is highly dependent on woodland, often not traveling far from roost to core foraging areas (Hill & Greenaway, 2006) and therefore may not be affected in the same way by the quality of the habitat surrounding a woodland, compared with more generalist and vagile species. The woodlands surveyed within this project were also all specifically selected to maximize the chance of encountering breeding female *M. bechsteinii* bats and therefore lower quality woodlands, where a higher proportion of males might be expected, were not surveyed. However, our results do indicate that resource partitioning does not occur in *M. bechsteinii*, with males and females both using high quality woodland habitat.

Our findings support key research in the area in highlighting the importance of isolated woodland patches in fragmented agricultural landscapes for bat species (Fuentes‐Montemayor et al., 2013). But importantly, we also show how the quality of a woodland's surrounding habitat can influence sex‐specific differences in use of the landscape. Bats have potential as bioindicator species in fragmented agricultural landscapes (Jones et al., 2009; Park, 2015; Russo et al., 2021). Studying bats in these habitats, allows ecological insights into the health and quality of key habitats that are important for a wide range of taxa. Therefore, management considerations for bat species in woodlands should consider the wider landscape perspective in order to maintain healthy viable populations.

## AUTHOR CONTRIBUTIONS

**Penelope Fialas:** Formal analysis (equal); investigation (equal); methodology (equal); writing – original draft (equal); writing – review and editing (equal). **Lia Gilmour:** Formal analysis (equal); project administration (equal); writing – original draft (equal); writing – review and editing (equal). **Sophie Vickress:** Formal analysis (supporting); investigation (supporting). **Emma Underwood:** Investigation (equal); methodology (equal). **Carol Williams:** Conceptualization (equal); funding acquisition (equal); methodology (equal); resources (equal). **Helen Miller:** Conceptualization (equal); funding acquisition (equal); methodology (equal); resources (equal). **Paul Lintott:** Conceptualization (equal); funding acquisition (equal); project administration (equal); supervision (lead); writing – review and editing (equal).

## Funding information

This project was funded by the University of the West of England and the Bat Conservation Trust.

## CONFLICT OF INTEREST

The authors declare no competing interests.

## Supporting information


Appendix S1
Appendix S2
Appendix S3
Appendix S4
Appendix S5
Appendix S6
Appendix S7
Appendix S8

## Data Availability

Data from the Bechstein's Bat Survey can be downloaded at a spatial resolution of 10 km from the National Biodiversity Network Atlas (https://doi.org/10.15468/a6jgbl). Finer resolution data are available on request from the Bat Conservation Trust, London, UK (www.bats.org.uk).
