# Supplementary material for: Sexual segregation occurs in bats within fragmented remnant woodlands in an agricultural landscape
Source: Ecol Evol. 2022 Oct 1;12(10):e9350. doi: 10.1002/ece3.9350 (PMC9526024; doi:10.1002/ece3.9350)
Supplement: Supplementary file 1 — Appendix S1 Appendix S2 Appendix S3 Appendix S4 Appendix S5 Appendix S6 Appendix S7 Appendix S8 [file ECE3-12-e9350-s001.docx]

Appendix 1: Selection criteria for woodlands included in the Bat Conservation Trust’s Bechstein’s Bat Survey (Miller, 2012).

| Selection criteria | Description |
| --- | --- |
| Overall woodland composition | Native broadleaf or mixed (>50% broadleaf). |
| Size | >25ha of continuous woodland, in a single block, or in two or three close stands of  well-connected woods |
| Canopy cover | High canopy, with at least 75% cover.  (Could be 50-74% cover if there is very  well-developed understory and species rich  herb layers). |
| Canopy composition | Predominantly native broadleaved  Species (>50%), preferably oak (*Quercus robur* and *Q. petraea*), ash (*Fraxinus excelsior*), or mixed including a high proportion of old oak. |
| Understory cover | Well developed with at least 50% cover |
| Understory composition | Native species, including hazel (*Corylus avellana*), hawthorn (*Crataegus monogyna* and *C. laevigata*) and/or holly (*Ilex aquifolium*). |

Appendix 2:

Additional considerations for woodland choice when more than one was available in a 10km sq.

| **Positive considerations** | **Negative considerations** |
| --- | --- |
| Presence of a small stream or pond within the woodland which retains water in  summer | Evidence of recent management to clear understory, remove older trees, or reinstate  coppicing |
| South-facing woods at lower elevations within the known range | Higher altitudes or excessive exposure within the known range |
| Evidence of woodpecker holes |  |
| Stands of mixed ages including trees of >100 years |  |
| Occurrence of other suitable woodlands across the wider landscape (20km sq.) |  |


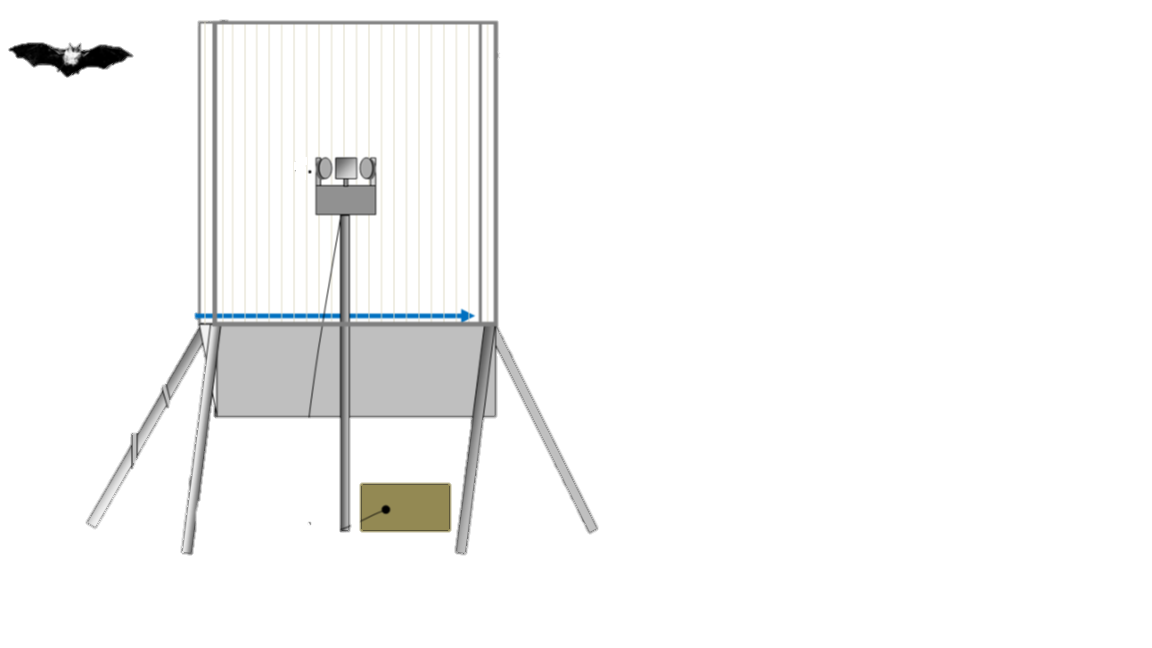


Appendix 3: Schematic of harp trap and lure setup; Sussex Autobat (a.) and associated speaker unit with rotating vane on pole (b.). Modified from Gilmour L., 2014 (*MSc dissertation-unpublished*).

**Appendix 4.** Mean and standard error of the amount (%) of each biotope in all six scales, across sites.

| **Biotope types** | 500m | 1km | 2km | 3km | 4km | 5km |
| --- | --- | --- | --- | --- | --- | --- |
| Unclassified | 0.07 (0.04) | 0.29 (0.14) | 0.67(0.25) | 1.05 (0.34) | 1.51 (0.41) | 1.78 (0.44) |
| Broadleaf woodland | 23.50 (0.72) | 18.44 (0.62) | 14.68 (0.57) | 13.08 (0.53) | 12.30 (0.49) | 11.83 (0.47) |
| Coniferous woodland | 3.06 (0.39) | 2.71 (0.37) | 2.47 (0.34) | 2.34 (0.28) | 2.31 (0.25) | 2.29 (0.23) |
| Arable land | 29.49 (0.39) | 32.46 (1.12) | 34.63 (1.09) | 34.97 (1.07) | 35.16 (1.02) | 35.23 (1.01) |
| Improved grassland | 30.78 (1.02) | 32.61(0.93) | 33.07 (0.88) | 33.06 (0.86) | 33.07 (0.81) | 32.94 (0.78) |
| Semi-natural habitat | 5.58 (0.37) | 5.98 (0.39) | 6.00 (0.37) | 5.97 (0.36) | 5.94 (0.34) | 5.97 (0.33) |
| Dwarf shrub heath | 0.84 (0.21) | 0.92 (0.23) | 1.06 (0.23) | 1.02 (0.20) | 0.99 (0.18) | 0.94 (0.17) |
| Mountain, bog | 0.31 (0.11) | 0.35 (0.10) | 0.35 (0.07) | 0.42 (0.11) | 0.46 (0.14) | 0.48 (0.14) |
| Saltwater | 0.40 (0.15) | 0.29 (0.13) | 0.31(0.13) | 0.37 (0.14) | 0.42 (0.15) | 0.49 (0.15) |
| Freshwater | 0.29 (0.05) | 0.28 (0.05) | 0.31(0.06) | 0.34 (0.06) | 0.39 (0.07) | 0.40 (0.07) |
| Costal habitat | 0.34 (0.15) | 0.33 (0.13) | 0.33 (0.11) | 0.39 (0.11) | 0.46 (0.11) | 0.53 (0.11) |
| Urban areas | 4.51 (0.65) | 5.32 (0.66) | 6.11 (0.63) | 6.53 (0.59) | 6.98 (0.56) | 7.12 (0.56) |

**Appendix 5**. Variance inflation factors (VIF) for final models of each response variable.

| Response variable | Predictor variable | VIF |
| --- | --- | --- |
| *P. auritus* | % Arable area (5km) | 1.14 |
|  | Connectivity to any woodland (1km) | 1.09 |
|  | Size (m^2^) | 1.04 |
|  | Date | 1.14 |
|  | Distance to nearest edge | 1.08 |
|  | Understory cover (%) | 1.36 |
|  | Canopy cover (%) | 1.27 |
|  |  |  |
| *M. nattereri* | % Improved grassland (5km) | 1.24 |
|  | Canopy cover (%) | 1.39 |
|  | Understorey cover (%) | 1.61 |
|  | Distance to nearest water | 1.13 |
|  | Distance to nearest edge | 1.17 |
|  | Mean temperature | 1.14 |
|  | Date | 1.26 |
|  | Shape index | 1.41 |
|  | Size (m^2^) | 1.35 |
|  |  |  |
| *M. mystacinus* | % Semi-natural area (1km) | 1.04 |
|  | Mean temperature | 1.04 |
|  |  |  |
| *M. bechsteinii* | Mean temperature | 1.07 |
|  | Understory cover (%) | 1.03 |
|  | % Freshwater (1km) | 1.06 |
|  |  |  |
| *P. pipistrellus* | % Coniferous woodland (3km) | 1.17 |
|  | Understorey cover (%) | 1.39 |
|  | Distance to nearest freshwater | 1.11 |
|  | Distance to nearest edge | 1.45 |
|  | Date | 1.32 |
|  | Canopy cover (%) | 1.23 |
|  |  |  |
| *P. pygmaeus* | % Freshwater (2km) | 1.03 |
|  | Canopy cover (%) | 1.28 |
|  | Distance to nearest freshwater | 1.02 |
|  | Understorey cover (%) | 1.19 |
|  | Date | 1.11 |
|  | Size (m^2^) | 1.30 |
|  | Shape index | 1.31 |

**Appendix 6.** Results of Mantel test performed to investigate spatial correlation (*r*) of the response variables. No correlation was found (empirical *P*-values > 0.05; 9999 permutations).

| Response Variable | \|r\| | *P* |
| --- | --- | --- |
| *M. nattereri* | 0.07 | 0.062 |
| *P. auritus* | 0.0 | 0.36 |
| *M. bechsteinii* | -0.02 | 0.54 |
| *P. pygmaeus* | 0.04 | 0.24 |
| *M. mystacinus* | -0.02 | 0.71 |
| *P. pipistrellus* | -0.01 | 0.50 |

**Appendix 7.** Standardized parameter estimates, standard error (SE), lower and upper 95% confidence intervals, *z* and *P* values and levels of significance (sig) of the variables present in the most parsimonious models (IT approach). Results give for effects of landscape and local characteristics on the probability of finding a female relative to a male (positive estimates refer to an explanatory variable that relates to an increased probability of finding a female).

| Response Variable | Independent Variable | Estimate | SE | Lower 95% CI | Upper 95% CI | *z* value | *P* | sig |
| --- | --- | --- | --- | --- | --- | --- | --- | --- |
| *P. auritus* | (Intercept) | -0.19 | 0.12 | -0.43 | 0.06 | 1.51 | 0.13 |  |
| *R^2^* 0.14 | Size (m2) | 0.16 | 0.14 | -0.03 | 0.45 | 1.16 | 0.24 |  |
|  | **% Arable area (5km)** | 0.43 | 0.14 | 0.15 | 0.71 | 3.00 | **0.00** | ** |
|  | **Connectivity to any Woodland (1km)** | 0.30 | 0.12 | 0.07 | 0.53 | 2.54 | **0.01** | * |
|  | Distance to nearest edge | -0.11 | 0.15 | -0.51 | 0.06 | 0.74 | 0.46 |  |
|  | Understorey cover (%) | 0.07 | 0.12 | -0.08 | 0.42 | 0.62 | 0.54 |  |
|  | Mean temperature | -0.04 | 0.09 | -0.41 | 0.11 | 0.41 | 0.68 |  |
|  | Canopy cover (%) | -0.01 | 0.05 | -0.43 | 0.11 | 0.18 | 0.86 |  |
|  | Date | 0.00 | 0.03 | -0.31 | 0.19 | 0.10 | 0.92 |  |
|  |  |  |  |  |  |  |  |  |
| *M. nattereri* | (Intercept) | -0.55 | 0.22 | -0.98 | -0.13 | 2.58 | 0.01 | * |
| *R^2^* 0.16 | Canopy cover (%) | 0.09 | 0.19 | -0.11 | 0.77 | 0.48 | 0.63 |  |
|  | **% Improved grassland (5km)** | 0.54 | 0.23 | 0.09 | 1.00 | 2.34 | **0.02** | * |
|  | Date | 0.27 | 0.25 | -0.04 | 0.82 | 1.05 | 0.29 |  |
|  | Shape | -0.30 | 0.25 | -0.80 | 0.03 | 1.22 | 0.22 |  |
|  | Understorey cover (%) | -0.25 | 0.29 | -0.94 | 0.10 | 0.86 | 0.39 |  |
|  | Mean temperature | 0.04 | 0.13 | -0.18 | 0.67 | 0.33 | 0.74 |  |
|  | Distance to nearest edge | 0.06 | 0.14 | -0.14 | 0.61 | 0.44 | 0.66 |  |
|  |  |  |  |  |  |  |  |  |
| *M. mystacinus* | (Intercept) | -0.84 | 0.39 | -1.60 | -0.07 | 2.13 | 0.03 | * |
| *R^2^* 0.29 | **Semi-natural area % (1km)** | -1.01 | 0.44 | -1.87 | -0.15 | 2.31 | **0.02** | * |
|  | Mean temperature | 0.17 | 0.29 | -0.23 | 1.03 | 0.58 | 0.56 |  |
|  |  |  |  |  |  |  |  |  |
| *M. bechsteinii* | (Intercept) | -0.23 | 0.53 | -1.26 | 0.80 | 0.44 | 0.66 |  |
| *R^2^* 0.59 | **% Freshwater (1km)^1^** | -1.58 | 0.79 | -3.13 | -0.03 | 2.00 | **0.05** | * |
|  | Mean temperature | -0.42 | 0.60 | -1.60 | 0.77 | 0.69 | 0.49 |  |
|  | Understorey cover (%) | 0.40 | 0.55 | -0.68 | 1.47 | 0.72 | 0.47 |  |
|  |  |  |  |  |  |  |  |  |
| *P. pipistrellus* | (Intercept) | -0.31 | 0.25 | -0.80 | 0.18 | 1.24 | 0.22 |  |
| *R^2^* 0.20 | **% Coniferous Woodland (3km)** | -0.57 | 0.30 | -1.17 | 0.03 | 1.87 | **0.06** | . |
|  | Understorey cover (%) | -0.25 | 0.30 | -0.98 | 0.10 | 0.83 | 0.41 |  |
|  | Distance to nearest freshwater | 0.15 | 0.31 | -0.28 | 1.22 | 0.49 | 0.63 |  |
|  | Distance to nearest edge | -0.06 | 0.18 | -0.96 | 0.31 | 0.30 | 0.76 |  |
|  | Date | 0.03 | 0.12 | -0.29 | 0.70 | 0.26 | 0.80 |  |
|  | Canopy cover (%) | -0.01 | 0.09 | -0.70 | 0.33 | 0.16 | 0.87 |  |
|  |  |  |  |  |  |  |  |  |
| *P. pygmaeus* | (Intercept) | -2.38 | 1.12 | -4.58 | -0.18 | 2.12 | 0.03 | * |
| *R^2^* 0.00 | % Freshwater (2km) | -2.75 | 2.80 | -8.59 | 2.33 | 0.98 | 0.33 |  |
|  | Canopy cover (%) | -0.22 | 0.31 | -1.04 | 0.09 | 0.71 | 0.48 |  |
|  | Distance to nearest freshwater | -0.31 | 0.63 | -2.41 | 0.69 | 0.49 | 0.62 |  |
|  | Understorey cover (%) | -0.07 | 0.22 | -1.19 | 0.27 | 0.32 | 0.75 |  |
|  | Date | 0.14 | 0.31 | -0.31 | 1.25 | 0.46 | 0.64 |  |
|  | Size (m^2^) | 0.06 | 0.23 | -0.45 | 1.38 | 0.26 | 0.80 |  |
|  | Shape | -0.05 | 0.22 | -1.38 | 0.47 | 0.25 | 0.80 |  |

^1^Significance disregarded due lack of variance and outlier influence (72% of sites had no water with this buffer)

**Appendix 8**. Results of the information theoretical or “IT” model averaging approach across a candidate set of models of GLMMs built to assess the effects of landscape and local characteristics on the probability of finding a female relative to a male. Only the most parsimonious models are shown (*ΔAICc*<2), ranked by the second order information criterion *AICc* values. Presented are the number of parameters (K), the small-samples Akaike Information Criterion (AICc) and AICc weight (Wt).

| **Response Variable** | **Model** | **K** | **AICc** | **ΔAICc** | **AICc Wt** |
| --- | --- | --- | --- | --- | --- |
|  |  |  |  |  |  |
| *M. nattereri* | Canopy cover (%) + % Improved grassland (5km) + Date + Shape index + Understorey cover (%) | 7 | 141 | 0.00 | 0.09 |
|  | % Improved grassland (5km) + Date + Shape index + Understorey cover (%) | 6 | 141 | 0.12 | 0.09 |
|  | % Improved grassland (5km) + Date + Shape index | 5 | 141 | 0.45 | 0.07 |
|  | % Improved grassland (5km) +Shape index | 4 | 141 | 0.60 | 0.07 |
|  | Canopy cover (%) + % Improved grassland (5km) + Date + Mean temperature + Shape index + Understorey cover (%) | 8 | 142 | 0.79 | 0.06 |
|  | % Improved grassland (5km) + Date + Distance to nearest edge + Shape index | 6 | 142 | 0.91 | 0.06 |
|  | % Improved grassland (5km) + Date + Distance to nearest edge + Shape index + Understorey cover (%) | 7 | 142 | 0.92 | 0.06 |
|  | % Improved grassland (5km) + Date + Understorey cover (%) | 5 | 142 | 1.12 | 0.05 |
|  | % Improved grassland (5km) + Date | 4 | 142 | 1.26 | 0.05 |
|  | Canopy cover (%) + % Improved grassland (5km) + Date + Distance to nearest edge+ Shape index + Understorey cover (%) | 8 | 142 | 1.28 | 0.05 |
|  | % Improved grassland (5km) | 3 | 142 | 1.38 | 0.05 |
|  | % Improved grassland (5km) + Date Mean temperature +  Shape index + Understorey cover (%) | 7 | 142 | 1.49 | 0.04 |
|  | % Improved grassland (5km) + Shape index + Understorey cover (%) | 5 | 142 | 1.50 | 0.04 |
|  | % Improved grassland (5km) + Distance to nearest edge + Shape index | 5 | 142 | 1.55 | 0.04 |
|  | % Improved grassland (5km) + Date + Distance to nearest edge | 5 | 143 | 1.84 | 0.04 |
|  | % Improved grassland (5km) + Mean temperature + Shape index | 5 | 143 | 1.86 | 0.04 |
|  | Canopy cover (%) + % Improved grassland (5km) + Shape index + Understorey cover (%) | 6 | 143 | 1.89 | 0.04 |
|  | Canopy cover (%) + % Improved grassland (5km) + Mean temperature + Shape index + Understorey cover (%) | 7 | 143 | 1.90 | 0.04 |
|  | % Improved grassland (5km) + Date + Distance to nearest edge + Understorey cover (%) | 6 | 143 | 1.98 | 0.03 |
|  |  |  |  |  |  |
| *M. mystacinus* | **Semi-natural area % (1km)** | 3 | 87 | 0.00 | 0.58 |
|  | Mean temperature + Semi-natural area % (1km) | 4 | 88 | 0.67 | 0.42 |
|  |  |  |  |  |  |
| *P. pipistrellus* | % Coniferous Woodland (3km) | 3 | 88 | 0.00 | 0.19 |
|  | % Coniferous Woodland (3km) + Understorey cover (%) | 4 | 88 | 0.08 | 0.18 |
|  | % Coniferous Woodland (3km) + Distance to nearest water + Understorey cover (%) | 5 | 88 | 0.49 | 0.15 |
|  | % Coniferous Woodland (3km) + Distance to nearest water | 4 | 89 | 1.37 | 0.09 |
|  | % Coniferous Woodland (3km) + Distance to nearest edge + Understorey cover (%) | 5 | 89 | 1.50 | 0.09 |
|  | % Coniferous Woodland (3km) + Distance to nearest water + Distance to nearest edge + Understorey cover (%) | 6 | 89 | 1.63 | 0.08 |
|  | % Coniferous Woodland (3km) + Date | 4 | 89 | 1.68 | 0.08 |
|  | Canopy cover (%) + % Coniferous Woodland (3km) | 4 | 89 | 1.81 | 0.08 |
|  | % Coniferous Woodland (3km) + Date + Understorey cover (%) | 5 | 89 | 1.81 | 0.08 |
|  |  |  |  |  |  |
| *P. pygmaeus* | % Freshwater (2km) | 3 | 69 | 0.00 | 0.10 |
|  | Canopy cover (%) + % Freshwater (2km) | 4 | 69 | 0.04 | 0.09 |
|  | % Freshwater (2km) + Distance to nearest water | 4 | 69 | 0.27 | 0.08 |
|  | % Freshwater (2km) | 4 | 69 | 0.74 | 0.07 |
|  | Canopy cover (%) + % Freshwater (2km) +Distance to nearest water | 5 | 69 | 0.79 | 0.06 |
|  | Canopy cover (%) + % Freshwater (2km) + Date | 5 | 70 | 1.04 | 0.06 |
|  | Size + Canopy cover (%) + % Freshwater (2km) | 5 | 70 | 1.27 | 0.05 |
|  | % Freshwater (2km) + Distance to nearest water + Understorey cover (%) | 5 | 70 | 1.28 | 0.05 |
|  | Canopy cover (%) +Date | 4 | 70 | 1.48 | 0.05 |
|  | % Freshwater (2km) + Distance to nearest water +Shape index | 5 | 70 | 1.56 | 0.04 |
|  | Canopy cover (%) + Date | 4 | 70 | 1.57 | 0.04 |
|  | % Freshwater (2km) + Shape index | 4 | 70 | 1.69 | 0.04 |
|  | Canopy cover (%) + % Freshwater (2km) +Date + Distance to nearest water | 6 | 70 | 1.70 | 0.04 |
|  | % Freshwater (2km) +Date + Distance to nearest water | 5 | 70 | 1.70 | 0.04 |
|  | Size + % Freshwater (2km) | 4 | 70 | 1.72 | 0.04 |
|  | Canopy cover (%) | 3 | 70 | 1.77 | 0.04 |
|  | Canopy cover (%) + Date + Distance to nearest water | 5 | 70 | 1.80 | 0.04 |
|  | % Freshwater (2km) +Date +Understorey cover (%) | 5 | 71 | 1.91 | 0.04 |
|  | Size + Canopy cover (%) + % Freshwater (2km) + Shape index | 6 | 71 | 1.97 | 0.04 |
|  |  |  |  |  |  |
| *M. bechsteinii* | % Freshwater (1km) + Mean temperature + Understorey cover (%) | 5 | 62 | 0.00 | 0.28 |
|  | % Freshwater (1km) | 3 | 62 | 0.17 | 0.26 |
|  | % Freshwater (1km) + Understorey cover (%) | 4 | 62 | 0.36 | 0.23 |
|  | % Freshwater (1km) + Mean temperature | 4 | 62 | 0.41 | 0.23 |
|  |  |  |  |  |  |
| *P. auritus* | Size (m2) + % Arable area (5km) + Connectivity to any Woodland (1km) + Distance to nearest edge | 6 | 332 | 0.00 | 0.14 |
|  | Size (m2) + % Arable area (5km) + Connectivity to any Woodland (1km) | 5 | 333 | 0.69 | 0.10 |
|  | Size (m2) + % Arable area (5km) + Connectivity to any Woodland (1km) + Distance to nearest edge + Understorey cover (%) | 7 | 333 | 0.77 | 0.09 |
|  | % Arable area (5km) + Connectivity to any Woodland (1km) | 4 | 333 | 0.84 | 0.09 |
|  | Size (m2) + % Arable area (5km) + Connectivity to any Woodland (1km) + Understorey cover (%) | 6 | 333 | 1.05 | 0.08 |
|  | Size (m2) + % Arable area (5km) + Connectivity to any Woodland (1km) + Mean temperature + Distance to nearest edge | 7 | 333 | 1.13 | 0.08 |
|  | % Arable area (5km) + Connectivity to any Woodland (1km) + Distance to nearest edge | 5 | 334 | 1.31 | 0.07 |
|  | Size (m2) + % Arable area (5km) + Connectivity to any Woodland (1km) + Mean temperature + Distance to nearest edge + Understorey cover (%) | 8 | 334 | 1.34 | 0.07 |
|  | % Arable area (5km) + Connectivity to any Woodland (1km) + Understorey cover (%) | 5 | 334 | 1.38 | 0.07 |
|  | Size (m2) + % Arable area (5km) + Connectivity to any Woodland (1km) + Mean temperature + Understorey cover (%) | 7 | 334 | 1.57 | 0.06 |
|  | Size (m2) + % Arable area (5km) + Connectivity to any Woodland (1km) + Mean temperature | 6 | 334 | 1.84 | 0.05 |
|  | Size (m2) + Canopy cover (%) + % Arable area (5km) + Connectivity to any Woodland (1km) + Understorey cover (%) | 7 | 334 | 1.90 | 0.05 |
|  | Size (m2) + % Arable area (5km) + Date+ Connectivity to any Woodland (1km) + Distance to nearest edge | 7 | 334 | 1.94 | 0.05 |
